# A Novel KPC Variant KPC-55 in *Klebsiella pneumoniae* ST307 of Reinforced Meropenem-Hydrolyzing Activity

**DOI:** 10.3389/fmicb.2020.561317

**Published:** 2020-10-07

**Authors:** Eun-Jeong Yoon, You Jeong Choi, Sun Hee Park, Jeong Hwan Shin, Sung Gyun Park, Jong Rak Choi, Seok Hoon Jeong

**Affiliations:** ^1^Department of Laboratory Medicine, Yonsei University College of Medicine, Seoul, South Korea; ^2^Research Institute of Bacterial Resistance, Yonsei University College of Medicine, Seoul, South Korea; ^3^Department of Infectious Disease Research, Busan Metropolitan City Institute of Health & Environment, Busan, South Korea; ^4^Department of Laboratory Medicine and Paik Institute for Clinical Research, Inje University College of Medicine, Busan, South Korea

**Keywords:** *Klebsiella pneumoniae*, KPC-55, meropenem, ST307, carbapenemase-producing Enterobacterales

## Abstract

A novel *Klebsiella pneumoniae* carbapenemase (KPC) variant, KPC-55, produced by a *K. pneumoniae* ST307 strain was characterized. *K. pneumoniae* strain BS407 was recovered from an active surveillance rectal swab of a patient newly admitted to a general hospital in Busan, South Korea. Carbapenemase production was confirmed by the modified Hodge test, and the MICs of β-lactams were determined by the broth microdilution method. The whole genome was sequenced. Cloning and expression of the *bla*_KPC–55_ gene in *Escherichia coli* and MIC determination were performed. The enzyme KPC-55 was used for kinetic assays against β-lactams and compared with the KPC-2 enzyme. The new allele of the *bla*_KPC_ gene had a T794A alteration compared to the *bla*_KPC–2_ gene, resulting in the amino acid substitution Y264N in the middle of the β9-sheet. Compared to the KPC-2-producing strain, the KPC-55-producing strain exhibited a lower level of resistance to most β-lactam drugs tested, however, the KPC-55 enzyme catalyzed aztreonam and meropenem at an increased efficiency compared to the catalytic activity of KPC-2. KPC subtypes could have varied phenotypes due to alterations in amino acid sequences, and such an unexpected resistance phenotype emphasizes the importance of detailed characterizations for the carbapenemase-producing Enterobacterales.

## Introduction

The β-lactam drugs are currently the most used class of antimicrobial agents; among them, carbapenems are the most potent against Gram-positive and Gram-negative bacteria and have the broadest spectrum of activity (Papp-Wallace et al., 2011). *Klebsiella pneumoniae* carbapenemase (KPC) is one of the most worrisome carbapenem resistance determinants in clinical settings, because it has broad spectrum of substrates including most β-lactams except cephamycins and it appears to be produced by a broad range of bacterial hosts (Yigit et al., 2001; Nordmann et al., 2011). The recent development regarding the inactivation of class A β-lactamases, including KPC, is the possibility to use avibactam in combination with ceftazidime (van Duin and Bonomo, 2016). However, emerging KPC subtypes resistant to avibactam is issuing (Gottig et al., 2019). To date, a total of 54 allele types of KPC have been deposited in the β-lactamase database^1^.

*Klebsiella pneumoniae* sequence type (ST) 307 was deposited into the multilocus sequence typing database primarily in 2008, and the ST307 strains are frequently resistant to late generation cephalosporins through its production of CTX-M-15 extended-spectrum β-lactamase (ESBL) (Wyres et al., 2019). In recent years, the spectrum of resistance of the *K. pneumoniae* ST307 clone was broadened to carbapenems by acquiring genes encoding carbapenemases, such as KPC (Villa et al., 2017). The KPC-producing *K. pneumoniae* clonal group (CG) has been changed from CG235 to ST307 in recent years (Yoon et al., 2018b; Cejas et al., 2019). In South Korea, most of the KPC-producing Enterobacterales were *K. pneumoniae* ST11 belonging to CG235 until 2014 and from 2015, the KPC-producing clone was rapidly exchanged to ST307 (Yoon et al., 2018b).

Here, we report a *K. pneumoniae* ST307 clinical strain recovered from an active surveillance rectal swab specimen against a new admission carrying an IncX3 plasmid, which encodes a novel subtype KPC-55 with asparagine at position 264 instead of tyrosine as in the prototype KPC-2. The amino acid substitution in the KPC-55 was responsible for the increased catalytic activity to meropenem compared to the catalytic activity of KPC-2.

## Materials and Methods

### Ethics

This study was approved by the Institutional Review Board of Inje University Busan Paik Hospital (No. 20-0036), Busan, South Korea.

### Clinical Strains Used in the Study

The *K. pneumoniae* strain BS407 was isolated from the CPE screening step before hospitalization using rectal swab of a patient in a university hospital in Busan, South Korea. The patient information was obtained retrospectively. Antibiograms were obtained by the disk diffusion test, and the carbapenemase-producing phenotype of the strain was confirmed by the modified Hodge test. For any comparison, the *K. pneumoniae* lab-collection strain KP1559, which harbors the prototype *bla*_KPC–2_ gene, was used.

### Antimicrobial Susceptibility Testing

An antibiogram for *K. pneumoniae* strain BS407 was performed by the disk diffusion test on Mueller-Hinton (MH) agar (Becton Dickinson, Franklin lakes, NJ, United States) following the CLSI guidelines (CLSI, 2019) with 15 antimicrobial agents, i.e., piperacillin, amoxicillin/sulbactam, cefazolin, cefotaxime, ceftazidime, cefepime, cefoxitin, aztreonam, ertapenem, imipenem, meropenem, amikacin, gentamicin, ciprofloxacin, and trimethoprim-sulfamethoxazole (Oxoid, Basel, Switzerland). The MICs of tigecycline (Pfizer, New York city, NY, United States), colistin (Sigma-Aldrich, St. Louis, MO, United States), and the eight β-lactam drugs (Table 1) were determined by the broth microdilution method using MH broth (CLSI, 2019). For ceftazidime and imipenem, MICs were determined with or without enzyme inhibitors, clavulanic acid (Dong-A Pharmaceutical Co., Ltd., Seoul, South Korea), and avibactam (BOC Sciences, Shirley, NY, United States). *Escherichia coli* ATCC 25922 and *Pseudomonas aeruginosa* ATCC 27853 were used for quality control of the tests.

**TABLE 1:** MICs of β-lactams.

	MICs (μg/ml)				
	
	*K. pneumoniae*		*E. coil*		
		
	BS407	KP1559	TOP10	TOP10	TOP10
				
β-Lactams^*a*^	Ω*bla*_KPC–55_	Ω*bla*_KPC–2_	Ω*bla*_KPC–55_	Ω*bla*_KPC–2_	
Ampicillin	>32	>32	>32	>32	4
Aztreonam	16	>32	16	>32	0.125
Cefotaxime	16	>32	1	8	<0.0625
Ceftazidime	16	>32	8	32	0.25
Ceftazidime + CA^*b*^	16	4	0.5	0.25	0.25
Ceftazidime + AB^*c*^	0.125	2	0.5	0.25	0.25
Cefoxitin	32	>32	2	>32	2
Cefepime	8	>32	1	8	<0.0625
Imipenem	1	8	0.5	1	0.125
Imipenem + CA	0.5	<0.0625	0.25	0.125	0.125
Imipenem + AB	<0.0625	<0.0625	0.5	0.25	0.25
Meropenem	2	16	0.25	1	<0.0625

*^*a*^Ampicillin (Sigma-Aldrich), aztreonam (Daehan New Pharm Co., Ltd., Gyeonggi-do, South Korea), cefotaxime (Aju Pharm Co., Ltd., Seoul, South Korea), ceftazidime (Sigma-Aldrich), cefoxitin (JW pharmaceutical, Co., Ltd., Seoul, South Korea), cefepime (Boryeong Pharmaceutical Co., Ltd., Seoul, South Korea), imipenem (MSD, Co., Inc., Kenilworth, NJ, United States), and meropenem (Sigma-Aldrich). ^*b*^CA, clavulanic acid (Dong-A Pharmaceutical Co., Ltd., Seoul, South Korea). ^*c*^AB, avibactam (BOC Sciences, Shirley, NY, United States).*

### Plasmid Transfer by Bacterial Conjugation

For bacterial conjugation, the *K. pneumoniae* BS407 strain was used as a donor, and a rifampicin-resistant mutant of *E. coli* J53 was used as a recipient. Equal amounts of exponential cultures of the donor, *K. pneumoniae* BS407, and recipient isolates were mixed, incubated in MH broth for 12 h, and spread on MH agar containing rifampicin (30 μg/ml), sodium azide (100 μg/ml), and imipenem (0.5 μg/ml). Any colony at the selective plate was tested by disk diffusion test and confirmed by PCR.

### DNA Manipulation and Cloning

The *bla*_KPC–2_ and *bla*_KPC–55_ genes were amplified from the total DNA of *K. pneumoniae* KP1559 and BS407, respectively, using KPC_F (5′-AGGAGGTAAATAATGTCACTGTATCGC CGTCTAGTT-3′) and KPC_R (5′-TTACTGCCCGTTGACGCCCAA-3′) using Phusion^®^ High-Fidelity DNA polymerase (Thermo Fisher Scientific, Waltham, MA, United States). Each PCR product was purified and cloned into the pCR-Blunt vector (Invitrogen, Thermo Fisher Scientific). The recombinant plasmids were transformed into chemically competent *E. coli* One Shot^TM^ TOP10 (Invitrogen, Thermo Fisher Scientific) and selected on MH agar containing kanamycin 50 μg/ml and ampicillin 50 μg/ml. Nucleic acid sequences and the direction of each insert were verified by Sanger sequencing using the universal M13 primers of both directions.

### Analysis of the Entire Genome

The whole genome of *K. pneumoniae* strain BS407 was sequenced using both Illumina and Nanopore technologies. DNA was extracted with the GenElute^TM^ Bacterial Genomic DNA Kit (Sigma), and libraries were prepared for Illumina using the Swift 2S Turbo DNA Library Kit (Swift Biosciences, Ann Arbor, MI, United States) and Swift 2S Turbo Combinatorial Dual Indexing Primer Kit (Swift Biosciences) and for Nanopore using the Ligation Sequencing Kit (Oxford Nanopore, Oxford, United Kingdom). Reads were assembled using Spades (version 3.11.1), and the complete sequences were annotated using prokka 1.13.7^2^. Identification of resistance determinants and plasmid incompatibility typing were assessed using ResFinder^3^ and plasmid finder^4^, respectively.

### Purification of the KPC Enzymes

*Klebsiella pneumoniae* BS407 and KP1559 cells were harvested by centrifugation, and the bacterial cells were resuspended in 15 mM sodium phosphate buffer (pH 7.0). The cells were disrupted by sonication, and the debris was eliminated by centrifugation at 15,000 × *g* for 30 min at 4°C. The crude extract was then passed through a 0.45-μm Millipore membrane filter (MilliporeSigma, Burlington, MA, United States) and loaded on a 10 ml Poly-Prep column (Bio-Rad, Hercules, CA, United States) filled with aminophenylboronic acid agarose (Sigma) at a flow rate of 1 ml/min (Bauvois et al., 2005). Then, the enzyme was eluted by a linear borate gradient (0 to 0.5 M) in 20 mM triethanolamine-HCl and 0.5 M NaCl (pH 7.0) over 5 (column volumes at a flow rate of 1 ml/min. The fractions exhibiting (-lactamase activity were collected, and their purity was estimated by electrophoresis on a sodium dodecyl sulfate-polyacrylamide gel stained with Bio-Safe Coomassie stain (Bio-Rad).

### Enzyme Kinetic Assay

Kinetic measurements were carried out using KPC enzymes in 50 mM morpholinepropanesulfonic acid (pH 7.0), 50 mM NaCl, and 100 mM sodium phosphate (pH 7.0) at 30°C (Bauvois et al., 2005). A Lambda 25 UV–visible (UV–Vis) spectrophotometer (PerkinElmer, Waltham, MA, United States) was used to determine the rates of hydrolysis. Various concentrations of the drugs were preincubated with the enzyme at 30°C to determine the kinetic parameters. All the values were determined in triplicate.

### Statistical Analysis

All kinetic results are presented as averages ± standard deviations from a minimum of three replicates.

### Accession Number

The genome sequence of the plasmid pBS407-3 was deposited in the United States National Center for Biotechnology Information (NCBI) database under the GenBank accession number MT028409, and the allele number of the *bla*_KPC–55_ gene was designated under the curation by the Bacterial Antimicrobial Resistance Reference Gene Database of the NCBI.

## Results

### Case Description

The *K. pneumoniae* BS407 strain was recovered from a rectal swab of >80-year-old patient admitted for hospitalization at Inje University Busan Paik Hospital, Busan, South Korea. The patient was subjected to active surveillance for carbapenemase-producing Enterobacterales (CPE) at the stage of admission.

The *K. pneumoniae* BS407 strain was resistant to ciprofloxacin and all β-lactam drugs tested, i.e., penicillins (ampicillin, piperacillin, and ampicillin-sulbactam with an inhibition zone diameter of 6 mm), narrow-spectrum (cefazoline, 6 mm) and extended-spectrum cephalosporins (cefotaxime, 18 mm; ceftazidime, 14 mm; and cefepime, 16 mm), cephamycin (cefoxitin, 14 mm), monobactam (aztreonam, 8 mm), and carbapenems (ertapenem, 15 mm; imipenem, 19 mm; and meropenem, 17 mm), but susceptible to aminoglycosides (amikacin, 22 mm; gentamicin, 24 mm), tigecycline (MIC, 2 μg/ml), and colistin (MIC, 2 μg/ml), and the strain showed intermediate resistance to trimethoprim-sulfamethoxazole (15 mm).

### Identification of a Novel KPC Subtype

**FIGURE 1:**
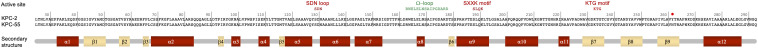
The KPC-55 protein in comparison with the prototype KPC-2. Top, the active site and binding site of the KPC enzyme corresponding to the 2OV5 protein database structure (Ke et al., 2007). Two hinge regions, the Ω loop and the substrate binding site, are indicated in the amino acid sequences in orange, green, and red, respectively. Middle, the sequence of KPC-55 compared with that of KPC-2 is presented. The Y264N alteration is illustrated with a red dot. Bottom, secondary structure of KPC-2 indicating β-sheets in light yellow and α-helices in dark red (Ke et al., 2007).

**FIGURE 2:**
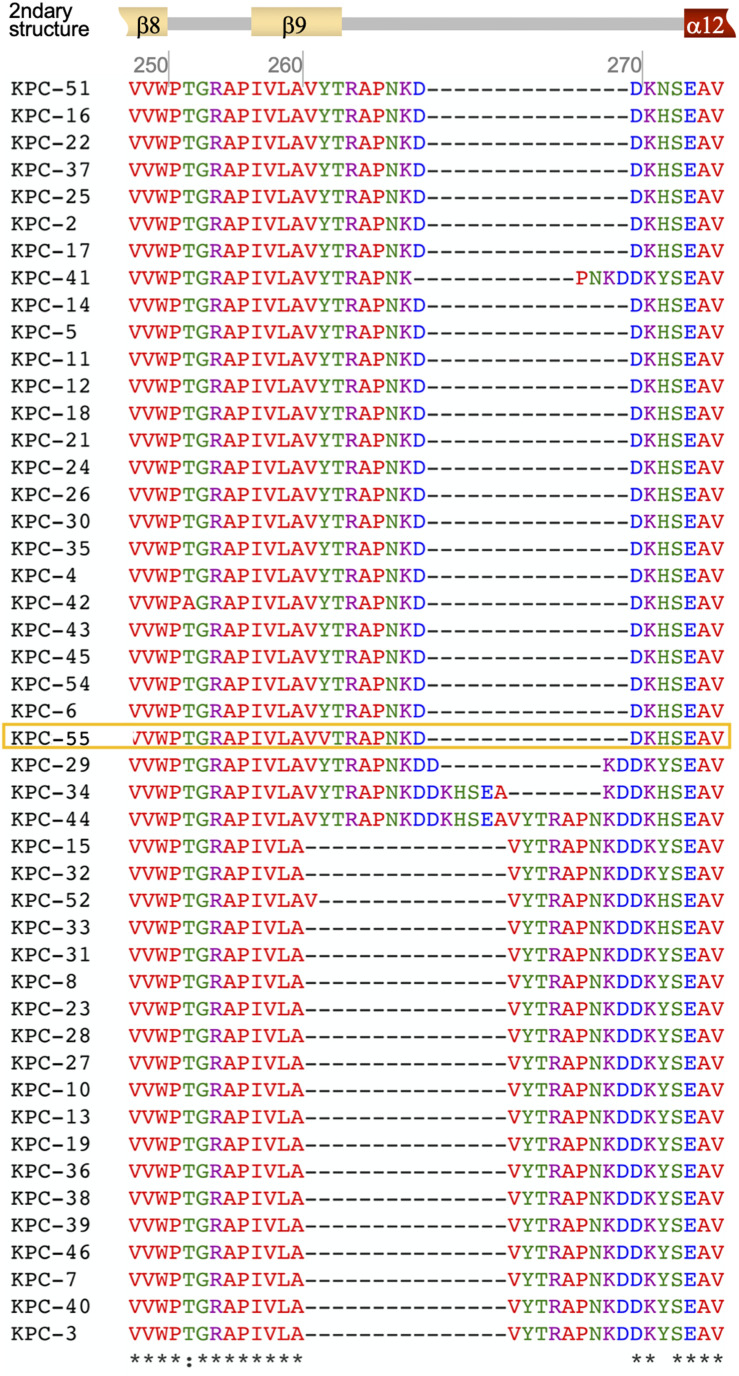
The variable region of the KPC subtypes between aa 250 and aa 278. The secondary structure of KPC-2 is indicated at the top with β-sheets in light yellow and alpha-helices in dark red. The amino acid sequence of KPC-55 is in the yellow open box. The sequences of the KPC subtypes were obtained from the Bacterial Antimicrobial Resistance Reference Gene Database ver. 2020-01-22.1 (BioProject accession, PRJNA313047; last accessed on 2020.2.5.).

Phenotypic verification using the modified Hodge test indicated that the BS407 strain was a carbapenemase producer, and PCR and direct sequencing confirmed that the strain harbored the *bla*_KPC_ gene, whose allele had never been deposited in the GenBank database. The novel gene encoding the new KPC variant had one nucleic acid alteration of T794A, resulting in asparagine instead of tyrosine at position 264 of the prototype KPC-2 (Figure 1). The altered amino acid was located in the middle of the β9-sheet, apart from the active site. Variable subtypes of KPC enzymes had one (KPC-52) to 15 aa (KPC-44) insertions between β9 and α12, 250 aa to 273 aa (Figure 2); however, the same substitution had not been identified previously.

### Genome Analysis of the *K. pneumoniae* BS407 Strain

The sequences of the whole genome of *K. pneumoniae* BS407 indicated that the strain belonged to ST307 and had the capsular type *wzi110*. The genome was composed of a 5,477,837-bp chromosome and four circularized plasmids of 136,158, 100,435, 50,505, and 3,551 bp in size. The intrinsic *bla*_SHV_ gene encoded SHV-28, an ESBL (Kim et al., 2006). In addition, the resistance determinants to fosfomycin, the *fosA* gene, was acquired in the chromosome, and the resistance-associated nucleic acid mutations was not observed in the chromosome, i.e., those at the quinolone resistance determining region or at the global regulator. The plasmids carried one to six resistance determinants, except the 3,551-bp cryptic plasmid: the *aac(6′)-Ib_cr_* and *qnrB1* genes for quinolone resistance, the *tet(A)* gene for tetracycline resistance, the *catB3* gene for chloramphenicol resistance, the *dfrA14* gene for trimethoprim resistance, and the *bla*_OXA–1_ gene for β-lactam resistance in the IncFIB-type 136,158-bp plasmid; the *qnrS1* gene for quinolone resistance in the IncFIB/FII-type 100,435-bp plasmid; and the *bla*_KPC–55_ and *bla*_SHV–182_ genes for β-lactam resistance in the IncX3-type 50,505-bp plasmid.

**FIGURE 3:**
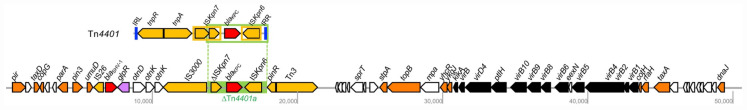
The 50,505-bp IncX3 plasmid carrying the *bla*_KPC–55_ gene in *K. pneumoniae* BS904. The open arrow indicates open reading frames, and the function of the protein encoded by the gene is represented by color: orange, replication and assembly; yellow, transposase; red, antimicrobial resistance; black, conjugation; white, others. The unlabeled ORF encodes a hypothetical protein. The green box indicates the truncated Tn*4401*. The complete structure of Tn*4401* is indicated at the top.

The *bla*_KPC–55_ gene was located on a truncated Tn*4401a* transposon composed of ΔIS*Kpn7*-*bla*_KPC–55_-IS*Kpn6* (Figure 3). The genes for transposase and resolvase composing the 3′ half of the transposon were absent together with the gene for transposase comprising the IS*Kpn7* element. The intergenic region between the IS*Kpn7* element and the *bla*_KPC–55_ gene was 99 bp shorter than the longest isotype Tn*4401b*, indicating that it was a Tn*4401a* element. The IncX3-type plasmid carrying the *bla*_KPC–55_ gene bracketed by the truncated Tn*4401a* element furnished the Type IV secretion system, contributing to conjugal DNA transfer (Figure 3). However, the transfer efficiency of the IncX3 plasmid carrying the *bla*_KPC–55_ gene was less than the detection limit, <10^–9^, which means that the plasmid was hardly transferable.

### Substrate Spectrum and Inhibitory Characteristics of KPC-55

To evaluate the spectrum of resistance, the corresponding *bla*_KPC–55_ gene was cloned into the pCR-Blunt vector and introduced into the *E. coli* OneShot TOP10 strain. When the gene was expressed in the *E. coli* host, the gene conferred reduced susceptibility to most β-lactams; however, the resulting MICs were consistently lower than the MICs with the *bla*_KPC–2_ gene (Table 1). As the *bla*_KPC–2_ transformant, the *bla*_KPC–55_ gene transformant presented diminished MICs of imipenem and ceftazidime by adding enzyme inhibitors, either clavulanic acid or avibactam.

Kinetic data using the enzyme showed that compared to that KPC-2, the hydrolytic activity of KPC-55 was lower for ampicillin and higher for aztreonam and meropenem (Table 2). The diminished catalytic efficiency for ampicillin was a result of the combination of the decreased turnover number and increased affinity. The higher catalytic efficiency of KPC-55 to aztreonam and meropenem was associated mostly with the improved turnover efficiency, not the affinity. It could be speculated that the alteration of Y264N could allow the enzyme to catalyze more meropenem and aztreonam but not ampicillin and increased the affinity of the enzyme to ampicillin. The catalytic efficiency of KPC-55 against imipenem and ceftazidime was indifferent from that of KPC-2, and KPC-55 presented similar levels of inhibition by clavulanic acid and avibactam to KPC-2.

**TABLE 2:** Kinetic parameters of the β-lactamases KPC-55 and KPC-2.

	Hydrolysis activity^*a*^					
	
	KPC-55			KPC-2		
		
Substrate	*k*_cat_ (s^–1^)	*K*_m_ (μM)	*k*_cat_/*K*_m_ (s^–1^ μM^–1^)	*k*_cat_ (s^–1^)	*K*_m_ (μM)	*k*_cat_/*K*_m_ (s^–1^ μM^–1^)
Ampicillin	0.06 ± 0.05	0.03 ± 0.01	1.89 ± 1.81	5.31 ± 2.8	1.03 ± 0.38	4.98 ± 0.77
Aztreonam	27.89 ± 5.20	1.50 ± 0.31	18.58 ± 0.36	2.56 ± 3.23	1.17 ± 1.29	1.85 ± 0.50
Ceftazidime	0.20 ± 0.33	0.40 ± 0.11	0.45 ± 0.74	0.02 ± 0.003	0.28 ± 0.09	0.07 ± 0.02
Imipenem	1.99 ± 0.64	0.13 ± 0.002	15.14 ± 4.61	0.41 ± 0.30	0.03 ± 0.02	37.48 ± 47.21
Meropenem	1.34 ± 0.05	0.02 ± 0.01	80.49 ± 35.18	0.09 ± 0.03	0.02 ± 0.02	5.19 ± 2.93

*^*a*^Data are the means of three independent determinations.*

## Discussion

The emergence of plasmid-mediated KPC enzymes in 1996 (Yigit et al., 2001) was a prelude of a global epidemic of CPE. The prototype KPC-2 is able to hydrolyze most β-lactams, with great efficiency for penicillins, cephalosporins and carbapenems and diminished efficiency for cephamycins and ceftazidime (Yigit et al., 2001). The identification of a number of KPC-2 variants possessing amino acid substitutions presented a varied spectrum of substrates for hydrolysis (Mehta et al., 2015). In particular, any alteration in the KPC active site, which is encompassed by the Ω-loop of R164 to D179, the hinge between helices α3 and α4 and another hinge region between helices α10 and α11, could affect the hydrolysis activity of the KPC enzyme (Galdadas et al., 2018). KPC-4 possessing the P103R substitution at the α3-α4 hinge has higher ceftazidime-hydrolytic activity but lower carbapenem-hydrolytic activity compared to KPC-2 (Wolter et al., 2009), and KPC-3 possessing the L169P alteration at the Ω-loop confers resistance to the bacterial hosts against the enzyme inhibitor avibactam, which inactivates KPC-2 (Hemarajata and Humphries, 2019). Not only the alterations located at the three loops but also the substitutions occurring at the other parts of the enzyme, such as the insertion of PNK between D269 and D270 in KPC-41, are responsible for reduced carbapenem-hydrolyzing activity and increased inhibition by avibactam (Mueller et al., 2019). All these examples highlight the importance of exploring new variants of the enzyme and characterizing them for their altered hydrolyzing activity.

In this study, a new allele of the KPC-2 variant with the Y264N alteration was characterized. The results indicate that the Y264N alteration was responsible for the reduced MICs of aztreonam, extended-spectrum cephalosporins, cefoxitin, and cefepime, and carbapenems in *E. coli* transformants presenting a good correspondence with the hydrolyzing activity test results using the enzyme. Interestingly, the alteration led to higher meropenem-hydrolyzing activity, even though the substitution was distantly located from the Ω-loop and any other hinges composing the active site. The β9-sheet and the following hinge to the α12-helix is a variable region; six of the 46 deposited KPC alleles have one- to 15-amino acid insertions in this region. Among those, only one allele, KPC-52, had an altered β9-sheet by insertion of valine between A259 and V260 (Figure 2); however, unfortunately, kinetic assay data for KPC-52 for proper comparison are unavailable.

The IncX3-type plasmid harboring the *bla*_KPC–55_ gene resembled the 69,409-bp plasmid pECSEV_01 in *E. coli* EcU443 recovered in 2014 from Seoul, South Korea (Jeong et al., 2018). They shared 99.9% nucleic acid identity showing 89% of query coverage. Of note, the reverse presented 100% of coverage including the duplicated conjugative elements in the pECSEV_01. The pECSEV_01 plasmid has been identified to harbor the *bla*_KPC–2_ gene carried by a truncated Tn*4401a* ΔIS*Kpn7*-*bla*_KPC–55_-IS*Kpn6* as the truncated Tn*4401a* harboring the *bla*_KPC–55_ gene.

The strain carrying the *bla*_KPC–55_ gene belonged to ST307, which is a worldwide *K. pneumoniae* clone resistant to extended-spectrum cephalosporins and carbapenems through varied acquired resistance determinants (Villa et al., 2017). The ST307 clone possessing the *bla*_KPC–2_ gene mobilized by the Tn*4401a* transposon has been predominant in South Korea (Kim et al., 2017; Yoon et al., 2018a), and it could be speculated that a point mutation occurred on the coding sequences of the *bla*_KPC–2_ gene, resulting in the BS407 strain. The *K. pneumoniae* ST307 clone is commonly resistant to multiple antimicrobial drugs but rarely harbors virulence factors (Kim et al., 2019). Because of its traits, the clone is linked with healthcare-associated infections (Kim et al., 2019) and associated with higher mortality than the other clones (Villa et al., 2017). Moreover, mortality in patients with infections caused by KPC-producing *K. pneumoniae* is high, up to 41.0% (Ramos-Castaneda et al., 2018), and even the carriage of KPC-producing *K. pneumoniae* is a risk factor for mortality in patients with diabetic foot disease (Tascini et al., 2015). Novel variants of the KPC enzyme have been reported in isolates identified not only from infection sites but also from active surveillance (Mueller et al., 2019). Therefore, following up both the dominant clonal lineage of KPC producers and the subtype of KPCs produced by Enterobacterales should be actively carried out both for infection-causative CPE and for carriages.

In conclusion, we reported here *K. pneumoniae* ST307 strain producing KPC-55, KPC-2_Y264N_, conferring reduced susceptibility to carbapenems. The alteration is located out of the known active site of the KPC enzyme, and a detailed kinetic assay revealed that the alteration led to higher meropenem-hydrolytic activity than the prototype KPC-2. KPC subtypes could have varied phenotypes by alterations, and the CPEs producing such an allele conferring low-level resistance could be missed in routine screening even though their catalytic activity toward some carbapenems can be improved. Thus, paying close attention to CPE screening is needed, and detailed characterization should be carried out.

## Data Availability Statement

The datasets presented in this study can be found in online repositories. The names of the repository/repositories and accession number(s) can be found in the article/supplementary material.

## Author Contributions

SHJ supervised the entire study and revised the manuscript. E-JY wrote the draft of the manuscript. YJC and E-JY performed the microbiology and biochemistry experiments and analyzed the data. SGP and JRC performed the whole genome sequencing, assembly, and annotation. JHS and SHP collected and identified the strain and new subtype subjected. All authors contributed to the article and approved the submitted version.

## Conflict of Interest

The authors declare that the research was conducted in the absence of any commercial or financial relationships that could be construed as a potential conflict of interest.

## Footnotes

1 http://www.bldb.eu/BLDB.php?prot=A#KPC, last updated in April 10, 2020

2 https://github.com/tseemann/prokka

3 https://cge.cbs.dtu.dk/services/ResFinder/

4 https://cge.cbs.dtu.dk/services/PlasmidFinder/
